# Examining the influence of Mother-in-law on family planning use in South Asia: insights from Bangladesh, India, Nepal, and Pakistan

**DOI:** 10.1186/s12905-023-02587-7

**Published:** 2023-08-09

**Authors:** Manas Ranjan Pradhan, Sourav Mondal

**Affiliations:** Department of Fertility and Social Demography, International Institute for Population Sciences (IIPS), Govandi Station Road, Deonar, Mumbai, Maharashtra 400088 India

**Keywords:** Family planning, Mother-in-law, Daughter-in-law, Co-residence, South Asia

## Abstract

**Background:**

Contraceptive use contributes to improved maternal and child health, education, empowerment of women, slow population growth, and economic development. The role of the family in influencing women’s health and health-seeking behavior is undergoing significant changes, owing to higher education, media exposure, and numerous government initiatives, in addition to women’s enhanced agency across South Asia. Against this backdrop, this study assesses the relationship between women’s living arrangements and contraceptive methods used in selected south Asian countries (India, Pakistan, Nepal, and Bangladesh).

**Methods:**

Data of currently married women aged 15–49 from the recent round of Demographic and Health Survey (DHS) of four South Asian countries, i.e., Nepal (2016), Pakistan (2017–18), Bangladesh (2017–18), and India (2019–21) had been used. Bivariate and multinomial logistic regression was performed using Stata with a 5% significance level.

**Results:**

Living arrangement of women had a significant association with contraceptive use in South Asia. The Mother-in-law (MIL) influenced the contraceptive method used by the Daughter-in-law (DIL), albeit a country-specific method choice. Modern limiting methods were significantly higher among women living with MIL in India. The use of the modern spacing method was considerably high among women co-residing with husband and/or unmarried child(ren) and MIL in Nepal and India. In Bangladesh, women living with husband and other family member including MIL were more likely to use modern spacing methods.. Women co-residing with the MIL had a higher likelihood of using any traditional contraceptive method in India.

**Conclusions:**

The study suggests family planning program to cover MIL for enhancing their understanding on the benefits of contraceptive use and modifying norms around fertility. Strengthening the interaction between the grassroots level health workers and the MIL, enhancing social network of DIL may help informed choice and enhance the use of modern spacing methods. Women’s family planning demands met with modern contraception, and informed contraceptive choices, must also be achieved to reach the 2030 Agenda for Sustainable Development.

## Background

Contraceptive use contributes to improved maternal and child health, education, empowerment of women, slow population growth, and economic development [1, 2]. Nevertheless, in South Asia, the use of modern contraception is still deficient, with an overall median contraceptive prevalence rate (CPR) of 37%. The unmet need for contraception is as high as 12% in the South Asian region [3]. Several dimensions of factors, i.e., individual characteristics (education, economic status, race, ethnicity, place of residence, religion, occupation), demographic factors (age, sex, parity), relationship characteristics (partnership types, communication, attitude), family or household characteristics (family structure, co-residence, household economy, division of labor), and community characteristics (cultural, social and political context) affect the sexual health of women including family planning [4]. Recent literature suggests that women’s age, education, place of residence, and region are the significant determinants of contraceptive use in Bangladesh, India, Nepal, and Pakistan [5–8]. Women’s occupation, body mass index, breastfeeding practice, husband’s education, wish for children, sexual activity in the past year, abstaining status, the number of children born in the last 5 years, and the total number of children ever died are significantly associated with contraceptive use in Bangladesh [5]. Social groups and the number of surviving sons are crucial factors of contraception use in India [8]. A strong positive impact of spousal communication on contraceptive use is found in Nepal [9]. The economic condition of women has been identified as a significant determinant of contraceptive use in India and Pakistan [6, 8].

Several studies have explored the individual determinants of contraceptive use, but very few have attempted to understand the relationship of contraceptive use with women’s living arrangements. The presence of a modern contraceptive user in the household positively influences the use of modern contraceptives among young married women [10]. The presence of mother-in-law (MIL) is found to be a significant influencer of contraceptive use of daughter-in-law (DIL) in South Asia [11]. Country-specific studies also indicate the contributory role of MIL in the reproductive health of the DIL, including contraception, such as in India [12–14], Pakistan [15, 16], Nepal [17] and Bangladesh [18]. In India, the MIL influences the modern-limiting method of contraception use of DIL but not of the modern spacing method of contraception use [13]. In Bangladesh, contraceptive use is high for a DIL with a MIL who supports contraceptive use [19].

The MIL influences DIL’s use of contraception sometimes directly, sometimes indirectly through her son [20] and sometimes by controlling the mobility [21] of DIL as well as restricting her from creating any peer group [12]. There again exist contradictory results in respect of modern contraceptive use of DIL by the presence or absence of MIL; some studies found the latter as a barrier [22] and some as a facilitator [13, 16, 23]. However, most of these past studies are (a) based on relatively smaller sample sizes, (b) do not represent the national scenario (c) focus only on modern methods without segregating limiting and spacing methods. South Asian societies are witnessing changes, as evident in the increased female education and work participation in secondary or tertiary sectors, the rise of youth culture that shapes the experience of new intimacies, amendments, and the enactment of new laws aimed at women empowerment [24]. Therefore, South Asia’s patriarchal family system is also compelled to adjust its norms and values. The role of the family in influencing women’s health and health-seeking behavior is, thus, undergoing significant changes, owing to higher education, media exposure, and numerous government initiatives, in addition to women’s enhanced agency across South Asia [24–26]. Against this backdrop, this study assesses the relationship between women’s living arrangements and contraceptive methods used in selected south Asian countries-India, Pakistan, Nepal, and Bangladesh.

## Methods

### Data

The most recent Demographic and Health Survey (DHS) data for four selected countries, i.e., Nepal (2016), Pakistan (2017–18), Bangladesh (2017–18), and India (2019–21), were used for the analysis. DHSs are nationally representative large-scale cross-sectional surveys that provide data on various health indicators, including contraceptive use. DHS sample designs are usually two-stage probability samples drawn from the most recent sample frame. The Indian DHS comprises two modules of questions, i.e., State and District modules. The sample of women covered in the state module is a subsample (15%) of the district module. Questions on husbands’ backgrounds and women’s work, women’s empowerment, HIV/AIDS, and domestic violence were administered to women in the state module only. However, DHS of remaining three countries contains only one module of questions representing the country. Informed consent procedures were followed in the DHSs, and only those who agreed voluntarily were interviewed. The surveys were approved by the Institutional Review Boards of the involved Institutes, and the datasets are freely available at https://www.dhsprogram.com. More details about DHS survey design, sampling, survey instruments, data collection procedure, and ethical considerations are present here [27]. The analysis was carried out for currently married women aged 15–49 in the selected countries, i.e., Bangladesh (*n* = 18,895), Nepal (9,897), Pakistan (11,902), and India (5,12,408).

### Outcome variable

The outcome variable used in this study was ‘contraceptive method use’; classified as not using any method, modern spacing methods, modern limiting methods, and any traditional method. Modern spacing methods include Pill, IUD, injections, diaphragm, condom, foam or jelly, lactational amenorrhea, and standard days. Female and male sterilization was considered a modern limiting method. Rhythm/ periodic abstinence and withdrawal were regarded as the traditional contraceptive methods.

### Predictor variables

The household living arrangement of the women was the primary predictor variable for this analysis. It was categorized as (1) Living with a husband and/or unmarried children: defined as households comprised of a married couple or a man or a woman living alone or with unmarried children with or without unrelated individuals; (2) Living with a husband and other family members except MIL: defined as households comprised of a married couple and other family members except MIL; (3) Living with husband and other family members including MIL: defined as household comprised of a married couple and other family members including the MIL, and (4), Living with a husband and/or unmarried children and MIL: defined as households comprised of a married couple or a man or a woman living alone or with unmarried children with or without unrelated individuals and with MIL.

To assess the adjusted effect of household living arrangements on contraceptive use, selected socioeconomic and demographic characteristics of the women such as current age (15–19, 20–24, 25–29, 30–34, 35–39, 40–44, 45–49), years of schooling (no schooling, less than 10 years, 10 and more years), desire for more children (no, yes, undecided), gender of living children (have only daughters, have at least one son), mass-media exposure (yes, no), wealth quintile (poorest, poorer, middle, richer, richest), religion (Hindu, Muslim, others-except for Pakistan), place of residence (rural, urban), geographical region were also included in the analysis.

### Statistical analysis

Univariate analysis was carried out to understand the profile of the study sample. Bivariate analysis was conducted to understand the individual association between the predictors and outcome variables. Multinomial logistic regression was used to check the adjusted effects of the predictor variables on contraceptive method use. The regression model used “not using any method” as the reference category. The predictor variables included for regression analysis were finalized after checking multicollinearity through the Variance Inflation Factors (VIF) test. Weights were used to restore the sample’s representativeness. The analyses were done with Stata (version 16) with a 5% significance level.

## Results

### Sample characteristics

Tables 1 and 2 presents the socioeconomic and demographic profile of the currently married women aged 15–49 in Bangladesh, Pakistan, India, and Nepal. Of the total women covered in the study, a majority were living in households with a husband and/or unmarried child(ren) in India (49%), Bangladesh (56%), and Nepal (48%). In Pakistan, most (45%) women lived in households with husbands and other family members except MIL. Most women had no schooling in Nepal (41%) and Pakistan (49%). The majority of women had no more desire for children in all four countries, i.e., India (74%), Nepal (73%), Bangladesh (64%), and Pakistan (47%). Close to three-fourths of women in all four countries had at least one living son, i.e., India (74%), Nepal (73%), Pakistan (73%), and Bangladesh (70%). India (82%) and Nepal (87%) had a majority of women of the Hindu religion, whereas, in Bangladesh, most (90%) followed Islam. In all four countries, the majority of women lived in rural areas, i.e., Bangladesh (64%), India (74%), Pakistan (73%), and Nepal (73%). Two-thirds of the women of Bangladesh (66%) and Pakistan (66%) had mass-media exposure. The corresponding figures were 76% in India and 82% in Nepal.

**Table 1:** Socioeconomic and demographic characteristics of currently married women aged 15–49, Bangladesh (2017–18) and Pakistan (2017–18)

Background characteristics	**Bangladesh**				**Pakistan**			
	**Unweighted %**	**Unweighted n**	**Weighted %**	**Weighted n**	**Unweighted %**	**Unweighted n**	**Weighted %**	**Weighted n**
**Household living arrangements of Women**								
Living with husband and/or unmarried child(ren)	54.99	10,391	55.63	10,562	42.77	5090	42.77	5060
Living with husband and other family member except MIL	31.37	5,928	31.47	5975	45.13	5,371	45.13	5339
Living with husband and other family member including MIL	11.68	2207	11.02	2092	10.8	1,286	10.8	1278
Living with husband and/or unmarried child(ren) and MIL	1.95	369	1.87	355	1.3	155	1.3	154
**Age**								
15–19	10.01	1,891	10.57	2006	5.01	596	5.01	592
20–24	17.96	3,394	18.09	3435	15.68	1,866	15.68	1855
25–29	18.16	3,431	18.14	3445	21.08	2,509	21.08	2494
30–34	17.43	3,293	17.43	3308	19.81	2,358	19.81	2344
35–39	14.56	2,751	14.22	2699	17.27	2,055	17.27	2043
40–44	11.23	2,122	11.11	2108	11.19	1,331	11.19	1323
45–49	10.65	2,013	10.44	1983	9.97	1,187	9.97	1180
**Years of schooling**								
No education	17.31	3271	18.08	3432	48.79	5,807	48.79	5773
< 10 years of schooling	63.87	12,069	64.70	12,284	27.35	3,256	27.35	3236
≥ 10 years of schooling	18.81	3555	17.22	3268	23.85	2,839	23.85	2822
**Desire for more children**^**a**^								
No	64.80	12,244	64.40	12,225	46.95	5582	46.95	5548
Yes	32.98	6231	33.39	6339	43.68	5193	43.68	5161
Undecided	2.22	420	2.21	420	9.37	1114	9.37	1107
**Gender of living children**								
Only have daughters	29.80	5,631	29.82	5661	27.36	3,256	27.36	3237
At least have one son	70.2	13,264	70.18	13,323	72.64	8,646	72.64	8594
**Mass media exposure**								
No	34.50	6,519	33.81	6419	33.77	4,018	33.77	3993
Yes	65.50	12,376	66.19	12,565	66.23	7,880	66.23	7831
**Religion**								
Hindu	9.31	1,760	8.64	1640				
Muslim	90.06	17,016	90.63	17,205				
Others	0.63	119	0.73	139				
**Wealth quintile**								
Poorest	18.77	3,547	18.29	3473	18.22	2,168	18.22	2155
Poorer	19.05	3,599	19.65	3730	19.42	2,311	19.42	2298
Middle	19.34	3,654	20.26	3846	20.35	2,422	20.35	2407
Richer	20.43	3,861	20.99	3985	20.92	2,490	20.92	2475
Richest	22.41	4,234	20.81	3951	21.10	2,511	21.10	2496
**Place of residence**								
Urban	36.42	6,881	28.33	5378	36.77	4,376	36.77	4350
Rural	63.58	12,014	71.67	13,606	63.23	7,526	63.23	7481
**Total**	**100**	**18,895**	**100**	**18,984**	**100**	**11,902**	**100**	**11,831**
**Region (Pakistan)**								
Punjab	53.06	6,315	53.06	6277				
Sindh	23.24	2,766	23.24	2750				
KPK	15.60	1,856	15.60	1845				
Balochistan	5.30	630	5.30	627				
IT	0.87	104	0.87	103				
FATA	1.93	230	1.93	229				
**Region (Bangladesh)**								
Barisal	10.65	2012	5.56	1056				
Chittagong	14.45	2731	17.98	3414				
Dhaka	14.94	2822	25.62	4864				
Khulna	13.13	2480	11.62	2205				
Mymensingh	10.87	2053	7.73	1468				
Rajshahi	12.84	2426	13.93	2645				
Rangpur	12.42	2346	11.84	2247				
Sylhet	10.72	2025	5.71	1085				

^a^Total number of women is different due to missing cases

**Table 2:** Socioeconomic and demographic characteristics of currently married women aged 15- 49, India (2019–21) and Nepal (2016)

**Background characteristics**	**India**				**Nepal**			
	**Unweighted %**	**Unweighted n**	**Weighted %**	**Weighted n**	**Unweighted %**	**Unweighted n**	**Weighted %**	**Weighted n**
**Household living arrangements of Women**								
Living with husband and or unmarried child	50.02	256,308	49.14	256,168	48.01	4,752	48.34	4771
Living with husband and other family member except MIL	39.00	199,840	39.39	205,344	43.77	4,332	43.08	4252
Living with husband and other family member including MIL	8.82	45,170	9.27	48,341	6.8	673	7.08	699
Living with husband and/or unmarried child(ren) and MIL	2.16	11,090	2.21	11,498	1.41	140	1.49	147
**Age of women**								
15–19	2.64	13,548	2.96	15,407	7.49	741	7.14	704
20–24	13.27	68,009	13.73	71,584	17.51	1,733	17.05	1682
25–29	19.61	100,506	19.61	102,257	19.62	1,942	19.83	1957
30–34	18.27	93,596	18.02	93,946	17.28	1,710	17.46	1723
35–39	17.74	90,897	17.39	90,684	15.29	1,513	15.29	1509
40–44	14.29	73,238	14.14	73,706	12.46	1,233	12.99	1282
45–49	14.17	72,614	14.15	73,767	10.36	1,025	10.24	1010
**Years of schooling**								
No education	28.84	147,753	27.57	143,754	41.86	4,143	41.53	4098
< 10 years of schooling	38.53	197,439	37.64	196,214	39.85	3,944	39.63	3910
≥ 10 years of schooling	32.63	167,216	34.79	181,384	18.29	1,810	18.85	1860
**Desire for more children**^**a**^								
No	72.13	362,966	73.71	377,038	73.44	7268	73.04	7208
Yes	23.67	119,096	23.16	118,469	24.42	2417	24.64	2432
Undecided	4.20	21,120	3.13	15,998	2.14	212	2.32	229
**Gender of living children**								
Only have daughters	25.13	128,776	25.87	134,890	25.88	2,561	26.45	2610
At least have one son	74.87	383,632	74.13	386,461	74.12	7,336	73.55	7258
**Mass Media Exposure**								
No	25.93	132,875	24.40	127,223	17.61	1,743	18.20	1796
Yes	74.07	379,533	75.60	394,129	82.39	8,154	81.80	8072
**Religion**								
Hindu	76.71	393,073	81.92	427,114	87.66	8,676	86.62	8547
Muslim	12.07	61,829	13.16	68,630	4.59	454	5.12	505
Others	11.22	57,506	4.91	25,607	7.75	767	8.27	816
**Wealth Quintile**								
Poorest	21.06	107,924	18.79	97,962	21.32	2,110	17.08	1685
Poorer	22.02	112,848	19.97	104,135	21.07	2,085	19.71	1945
Middle	20.74	106,285	20.43	106,487	20.79	2,058	21.14	2086
Richer	19.18	98,260	20.76	108,247	19.80	1,960	21.35	2106
Richest	17.00	87,091	20.05	104,520	17.02	1,684	20.73	2045
**Place of residence**								
Rural	76.18	390,362	68.66	357,957	63.24	6,259	61.06	6025
Urban	23.82	122,046	31.34	163,394	36.76	3,638	38.94	3843
**Total**	**100**	**512,408**	**100**	**521,352**	100	**9,897**	100	**9868**
**Region (India)**								
North	19.92	102,094	13.73	71,597				
Central	22.86	117,128	23.64	123,236				
East	17.12	87,731	23.83	124,256				
Northeast	13.78	70,596	3.67	19,144				
West	10.26	52,592	14.34	74,774				
South	16.05	82,267	20.78	108,344				
**Region (Nepal)**								
Province 1	14.11	1,396	16.76	1654				
Province 2	17.72	1,754	21.96	2167				
Province 3	11.83	1,171	19.45	1920				
Province 4	12.27	1,214	9.62	949				
Province 5	16.01	1,585	17.70	1747				
Province 6	14.34	1,419	5.94	586				
Province 7	13.72	1,358	8.56	845				

^a^Total number of women is different due to missing cases

### Current contraceptives use by household living arrangements and socio-demographic factors

Tables 3 and 4 presents the current use of contraceptive methods among currently married women aged 15–49 in Bangladesh, Pakistan, India, and Nepal by living arrangements of women and other socio-demographic characteristics. In Bangladesh, the use of the contraceptive method was 66% among women living with husbands and/or unmarried children, 52% for women living with husbands and other family members excluding MIL, 67% for women living with husbands and other family members, including MIL, and 65% for women living with husbands and/or unmarried children and MIL. The corresponding figures were- 40%, 28%, 34%, and 41% in Pakistan; 71%, 61%, 69%, and 71% in India; and 58%, 45%, 60%, and 67% in Nepal.

**Table 3:** Current use of the contraceptive method among currently married women aged 15–49 by socioeconomic and demographic characteristics, Bangladesh (2017–18) and Pakistan (2017–18)

Background characteristics	**Bangladesh**						**Pakistan**					
	**Not using**	**Modern Spacing method**	**Modern Limiting method**	**Any Traditional method**	**No. of women (UW)**	**No. of women (W)**	**Not using**	**Modern Spacing method**	**Modern Limiting method**	**Any Traditional method**	**No. of women (UW)**	**No. of women (W)**
**Household living arrangements of Women**												
Living with husband and/or unmarried child(ren)	33.9	48.6	6.9	10.7	10,391	10,562	59.6	17.1	12.5	10.8	5090	5060
Living with husband and other family member except MIL	47.9	38.3	4.7	9.2	5,928	5975	71.7	14.3	5.7	8.4	5,371	5339
Living with husband and other family member including MIL	32.5	53.6	4.7	9.2	2,207	2092	66.8	18.9	7.3	7.0	1,286	1278
Living with husband and/or unmarried child(ren) and MIL	34.6	54.6	4	6.8	369	355	58.5	30.8	8.1	2.6	155	154
**Age**												
15–19	51.1	43.6	0.1	5.2	1,891	2006	92.6	5.9	0	1.5	596	592
20–24	44.4	50.4	0.5	4.7	3,394	3435	81.7	13.1	0.2	5	1,866	1855
25–29	36.5	53.5	3.3	6.7	3,431	3445	71.6	17.5	3.4	7.5	2,509	2494
30–34	29	55.2	7.4	8.3	3,293	3308	57.9	21.8	8.4	12	2,358	2344
35–39	24.6	50.5	11	13.9	2,751	2699	55.9	19.8	12.8	11.4	2,055	2043
40–44	33.8	34.9	11.1	20.2	2,122	2108	52.3	14.7	21.3	11.6	1,331	1323
45–49	55.4	18.2	10.6	15.8	2,013	1983	63.4	7.8	18.1	10.8	1,187	1180
**Years of schooling**												
No education	37.2	37.1	11.9	13.8	3271	3432	71.4	12	9.6	7	5,807	5773
< 10 years of schooling	37.7	48.24	5.13	8.93	12,069	12,284	63.6	18	9	9.4	3,256	3236
≥ 10 years of schooling	40.8	46.96	2.5	9.74	3555	3268	57	22.8	7	13.2	2,839	2822
**Desire for more children**^**a**^												
No	30.8	47.6	9.15	12.37	12,244	12,225	46.49	21.45	18.79	13.27	5582	5548
Yes	51.23	43.29	0	5.48	6231	6339	83.32	11.18	0	5.50	5193	5161
Undecided	53.02	40.03	0	6.95	420	420	81.03	13.29	0	5.69	1114	1107
**Gender of living children**												
Only have daughters	52.45	38.53	1.99	7.03	5,631	5661	89.86	6.85	0.36	2.93	3,256	3237
At least have one son	32.07	49.18	7.55	11.19	13,264	13,323	56.77	19.73	12	11.5	8,646	8594
**Mass media exposure**												
No	39	44	5.9	11	6,519	6419	74.7	12	7.3	6	4,018	3993
Yes	37.7	47	5.9	9.4	12,376	12,565	61.3	18.3	9.6	10.8	7,880	7831
**Religion**												
Hindu	28.06	50.57	8.76	12.6	1,760	1640						
Muslim	39.19	45.5	5.65	9.66	17,016	17,205						
Others	28.37	55.06	1.71	14.87	119	139						
**Wealth quintile**												
Poorest	33.7	49.9	7.3	9.1	3,547	3473	79.9	9.9	7.2	3	2,168	2155
Poorer	36.5	46.1	6.6	10.9	3,599	3730	71	13.4	9.2	6.4	2,311	2298
Middle	40.8	44.3	5.9	8.9	3,654	3846	63.3	17.7	9.2	9.8	2,422	2407
Richer	38.9	45.9	5.4	9.8	3,861	3985	61.6	18.5	9.1	10.8	2,490	2475
Richest	40.2	44.3	4.5	11	4,234	3951	55.5	20.5	9.2	14.8	2,511	2496
**Place of residence**												
Urban	34.6	49.6	5.3	10.5	6,881	5378	57.5	19	9.8	13.7	4,376	4350
Rural	39.6	44.6	6.1	9.7	12,014	13,606	70.6	14.6	8.2	6.5	7,526	7481
**Total**	**38.1**	**46**	**5.9**	**10**	**18,895**	**18,984**	**65.8**	**16.2**	**8.8**	**9.2**	**11,902**	**11,831**
**Region (Pakistan)**												
Punjab	61.7	16.6	10.6	11.1	6,315	6277						
Sindh	69.1	14.5	10	6.5	2,766	2750						
KPK	69.1	19.1	4	7.7	1,856	1845						
Balochistan	80.2	11.7	2.4	5.8	630	627						
IT	54.3	25.2	9.5	11	104	103						
FATA	78.2	12.7	1	8.1	230	229						
**Region (Bangladesh)**												
Barisal	38.4	47.9	3.1	10.7	2012	1056						
Chittagong	46.3	40.5	4.3	9	2731	3414						
Dhaka	37.8	46.7	5.9	9.5	2822	4864						
Khulna	35.4	46.1	6.1	12.5	2480	2205						
Mymensingh	36.6	50.4	4.7	8.2	2053	1468						
Rajshahi	35.3	47.9	7.2	9.6	2426	2645						
Rangpur	30.2	50.3	8.6	10.8	2346	2247						
Sylhet	44.6	38.9	5.9	10.6	2025	1085						

^a^Total number of women is different due to missing cases, UW means ‘Unweighted’, W means ‘Weighted’

**Table 4:** Current use of the contraceptive method among currently married women aged 15–49 by socioeconomic and demographic characteristics, India (2019–21) and Nepal (2016)

Background characteristics	**India**						**Nepal**					
	**Not using**	**Modern spacing method**	**Modern limiting method**	**Any traditional method**	**No. of women (UW)**	**No. of women (W)**	**Not using**	**Modern spacing method**	**Modern limiting method**	**Any traditional method**	**No. of women (UW)**	**No. of women (W)**
**Household living arrangements of Women**												
Living with husband and or unmarried child	28.9	16.5	44.6	10	256,308	256,168	42.1	24.2	23.2	10.4	4,752	4771
Living with husband and other family member except MIL	39.4	20.3	29.7	10.6	199,840	205,344	55.1	19.4	16.5	8.9	4,332	4252
Living with husband and other family member including MIL	31.4	18.1	40.1	10.3	45,170	48,341	39.5	28.3	21.2	11	673	699
Living with husband and/or unmarried child(ren) and MIL	29.2	20	39.8	11	11,090	11,498	32.7	33.6	23	10.6	140	147
**Age of women**												
15–19	71.9	18.4	0.4	9.4	13,548	15,407	76.9	14.5	0	8.6	741	704
20–24	57.5	23.9	7.9	10.7	68,009	71,584	68	21.3	2.7	8.1	1,733	1682
25–29	39.1	24.8	24.6	11.5	100,506	102,257	54.2	27.2	9.8	8.8	1,942	1957
30–34	26.4	22.3	40	11.2	93,596	93,946	41.5	27.1	20.3	11.2	1,710	1723
35–39	21.1	17.3	50.8	10.8	90,897	90,684	31.5	24.3	33.2	11.1	1,513	1509
40–44	22.8	11.2	56.1	9.8	73,238	73,706	30.6	21.1	37.3	11	1,233	1282
45–49	27.9	6.5	58.7	6.9	72,614	73,767	34.7	13.3	42.2	9.8	1,025	1010
**Years of schooling**												
No education	29.9	11.2	48.9	10	147,753	143,754	42	20	31.5	6.5	4,143	4098
< 10 years of schooling	31.3	17.7	40.9	10.1	197,439	196,214	52.5	23.5	14.2	9.8	3,944	3910
≥ 10 years of schooling	38.2	24.3	26.9	10.6	167,216	181,384	48.5	26.5	7.9	17.1	1,810	1860
**Desire for more children**^**a**^												
No	21.07	16.12	52.85	9.97	362,966	377,038	37.46	24.99	27.64	9.92	7268	7208
Yes	63.35	25.06	0	11.6	119,096	118,469	75.09	15.27	0	9.63	2417	2432
Undecided	57.58	28.65	0	13.77	21,120	15,998	66.06	25.17	0	8.77	212	229
**Gender of living children**												
Only have daughters	58.1	19.8	12.4	9.6	128,776	134,890	71.71	16.81	1.82	9.66	2,561	2610
At least have one son	24.6	17.7	47.2	10.5	383,632	386,461	38.65	24.68	26.79	9.88	7,336	7258
**Mass Media Exposure**												
No	37.7	15.5	35.3	11.5	132,875	127,223	53.9	19.5	21	5.6	1,743	1796
Yes	31.9	19.1	39.2	9.9	379,533	394,129	45.9	23.3	20	10.8	8,154	8072
**Religion**												
Hindu	32.1	17.1	40.9	9.9	393,073	427,114	45.95	22.36	21.54	10.15	8,676	8547
Muslim	39.8	25.4	21.9	12.8	61,829	68,630	69.61	14.16	10.28	5.95	454	505
Others	35.2	18.5	36.8	9.5	57,506	25,607	48.77	30.3	12.2	8.73	767	816
**Wealth Quintile**												
Poorest	37.8	15.9	34.8	11.5	107,924	97,962	50.9	28.1	13.7	7.3	2,110	1685
Poorer	33.9	16.8	38.7	10.6	112,848	104,135	46.6	23.5	21.3	8.6	2,085	1945
Middle	32.3	15.7	42.6	9.4	106,285	106,487	50.4	18.2	24.4	7	2,058	2086
Richer	32.2	17.7	40.8	9.3	98,260	108,247	49.9	19.8	21.9	8.4	1,960	2106
Richest	30.6	25	33.7	10.7	87,091	104,520	39.7	24.6	18.4	17.4	1,684	2045
**Place of residence**												
Rural	34.4	16.5	39	10	122,046	357,957	45.2	23.7	20.5	10.6	6,259	6025
Urban	30.7	22	36.5	10.7	390,362	163,394	50.8	20.9	19.7	8.6	3,638	3843
**Total**	**33.3**	**18.2**	**38.2**	**10.3**	**512,408**	**521,352**	**47.4**	**22.6**	**20.2**	**9.8**	**9,897**	**9868**
**Region (India)**												
North	28.8	25.4	33.5	12.4	102,094	71,597						
Central	34.8	22.6	28.7	13.9	117,128	123,236						
East	34	19.5	32.2	14.3	87,731	124,256						
Northeast	40.7	33.8	9.2	16.2	70,596	19,144						
West	34.1	15.5	44.9	5.5	52,592	74,774						
South	31.9	6.2	59.5	2.3	82,267	108,344						
**Region (Nepal)**												
Province 1	44.9	27.2	12.9	15	1,396	1654						
Province 2	52.3	9.8	32.4	5.5	1,754	2167						
Province 3	39.4	30.4	18.8	11.4	1,171	1920						
Province 4	51.6	19.5	17.8	11.1	1,214	949						
Province 5	52	23.4	15.4	9.1	1,585	1747						
Province 6	48.9	27	17.5	6.6	1,419	586						
Province 7	48.9	27	17.5	6.6	1,358	845						

*UW* means ‘Unweighted’, *W* means ‘Weighted’

^a^Total number of women is different due to missing cases

Modern spacing method use varied by household living arrangements of women across the four selected south Asian countries. The prevalence of modern spacing method use was higher among women from households with a MIL except other family members than those residing in other types of households in all countries except India. For example- in Bangladesh, 55% of those from households with a MIL except other family members were using any modern spacing method and 54% of those from households with a MIL and with other family members were using any modern spacing method, which was 38% among women from households without the MIL, 49% for women living in households comprising husbands and/or unmarried children. Across the countries, the use of modern limiting methods of contraception was the highest among women living in households comprising husbands and/or unmarried children only than their counterparts from households with/without MIL. Between the women from households with and without MIL, modern method use was considerably higher among those from households with MIL. The result is true, except for Bangladesh, where modern limiting method use was almost the same (4.6%-4.7%) for women from households with/without the MIL. No specific pattern emerged so far as the use of traditional contraception methods was concerned, ranging between 7–11% for women with different living arrangements in Bangladesh, India, and Nepal. In Pakistan, 3% of the women from households with a MIL except other family members and 7% of the women from households with a MIL including other family members were using any traditional contraceptive method, which was 11% for women living in households comprising husbands and/or unmarried children.

Across the countries, the use of modern limiting methods increased with the increasing age of women. However, a higher prevalence of modern spacing methods was noticed for women aged 20–34, after which it declined. In all four countries, the years of schooling of women were positively associated with the use of the modern spacing method and inversely with the use of the modern limiting method. Higher percentages of the women with at least one surviving son were using any modern limiting method than those with only surviving daughters. Higher percentages of the women with mass-media exposure were using any modern spacing and limiting method of contraception in all four countries compared to those without mass-media exposure. The only exception was the use of modern limiting methods in Nepal. With the increasing wealth status of women, the use of the modern spacing method of contraception increased in India and Pakistan. Again, except in Pakistan, the use of modern limiting methods decreased with increasing wealth status. Contraceptive use pattern further varied by geographic region in all four selected countries. A sizable percentage of the women not desiring more children were not using any contraceptive methods across the four countries.

### Determinants of current use of contraceptive methods

Table 5 presents the adjusted odds ratio (AOR) of logistic regression of factors affecting the current use of contraceptive methods. In Nepal, the odds of using modern spacing methods of contraception were significantly higher among women living with husband and/or unmarried children and MIL (OR = 2.35, CI = 1.53–3.60) as well as those living with husband and other family members, including MIL (OR = 1.63, CI = 1.32–2.02) than those women living with only husband and/or unmarried children. Similarly, the women co-residing with husband and other family member including MIL had higher probability of using any modern spacing method than their counterparts co-residing with only husband and/or unmarried children in Bangladesh (OR = 1.17, CI = 1.05–1.31). The chances of using any modern spacing methods of contraception were significantly higher among the women living in households including husband and/or unmarried children and MIL (OR = 1.12, CI = 1.05–1.18) than their counterparts living in households with only a husband and/or unmarried child(ren) in India. The chances of using any modern limiting methods of contraception were significantly higher among the women living in households including husband and/or unmarried children and MIL (OR = 1.20, CI = 1.13–1.27) as well as households including husband and other family members including MIL (OR = 1.12, CI = 1.09–1.16) than their counterparts living in households with only a husband and/or unmarried child(ren) only in India. As against the women from households with husbands and/or unmarried children, those living in households with husband and/or unmarried children and MIL (OR = 1.13, CI = 1.06–1.21) as well as with husband and other members including MIL (OR = 1.07, CI = 1.03–1.11) had higher odds of using any traditional method of contraception in India.

**Table 5:** Adjusted Odds Ratio (AOR) of predictors of the contraceptive method use in selected South Asian countries

Background characteristics	Modern Spacing Method AOR (95% CI)				Modern limiting Method AOR (95% CI)				Any traditional Method AOR (95% CI)			
	Nepal	Pakistan	Bangladesh	India	Nepal	Pakistan	Bangladesh	India	Nepal	Pakistan	Bangladesh	India
**Household living arrangements of Women**												
Living with husband and or unmarried child^a^												
Living with husband and other family member except MIL	0.79*** [0.70, 0.89]	0.83** [0.75, 0.93]	0.70*** [0.65, 0.76]	0.92*** [0.90, 0.93]	0.94 [0.82, 1.07]	0.95 [0.81, 1.12]	0.81** [0.69, 0.94]	0.87*** [0.85, 0.88]	0.85 [0.73, 1.00]	0.84 [0.73, 0.96]	0.82*** [0.73, 0.92]	0.92*** [0.90, 0.94]
Living with husband and other family member including MIL	1.63*** [1.32, 2.02]	1.05 [0.90, 1.23]	1.17** [1.05, 1.31]	1 [0.97, 1.03]	1.33 [1.03, 1.72]	0.9 [0.69, 1.17]	0.99 [0.79, 1.25]	1.12*** [1.09, 1.16]	1.33 [0.99, 1.79]	0.83 [0.67, 1.03]	1.08 [0.90, 1.28]	1.07*** [1.03, 1.11]
Living with husband and/or unmarried child(ren) and MIL	2.35*** [1.53, 3.60]	1.49 [1.04, 2.13]	1.13 [0.89, 1.43]	1.12*** [1.05, 1.18]	1.6 [0.92, 2.78]	0.92 [0.44, 1.93]	0.7 [0.38, 1.29]	1.20*** [1.13, 1.27]	1.63 [0.88, 3.03]	0.83 [0.47, 1.49]	0.93 [0.62, 1.41]	1.13*** [1.06, 1.21]
**Age of women**												
15–19	1.75*** [1.26, 2.43]	2.86*** [1.94, 4.21]	9.16*** [7.62, 11.02]	1.93*** [1.82, 2.05]	0 [0.00, .]	0 [0.00, .]	0.42 [0.10, 1.72]	0.03*** [0.02, 0.05]	0.77 [0.51, 1.17]	1.27 [0.75, 2.15]	0.93 [0.69, 1.26]	0.99 [0.92, 1.06]
20–24	1.93*** [1.49, 2.51]	4.10*** [3.19, 5.28]	8.12*** [6.90, 9.55]	2.50*** [2.40, 2.60]	0.13*** [0.09, 0.19]	0.10*** [0.03, 0.32]	0.41*** [0.25, 0.69]	0.33*** [0.31, 0.34]	0.52*** [0.37, 0.72]	1.56** [1.16, 2.11]	0.78 [0.61, 0.98]	1.13*** [1.08, 1.18]
25–29	2.02*** [1.59, 2.56]	4.38*** [3.50, 5.49]	7.39*** [6.33, 8.62]	3.07*** [2.96, 3.18]	0.29*** [0.23, 0.37]	0.66** [0.49, 0.90]	1.18 [0.90, 1.54]	0.71*** [0.69, 0.73]	0.54*** [0.40, 0.73]	1.68*** [1.31, 2.17]	0.97 [0.79, 1.19]	1.47*** [1.42, 1.53]
30–34	2.20*** [1.73, 2.79]	4.53*** [3.64, 5.64]	7.67*** [6.58, 8.92]	3.66*** [3.53, 3.80]	0.60*** [0.48, 0.74]	1.11 [0.88, 1.41]	2.37*** [1.90, 2.95]	1.12*** [1.09, 1.15]	0.96 [0.72, 1.28]	2.17*** [1.72, 2.75]	1.26 [1.04, 1.52]	1.92*** [1.84, 1.99]
35–39	2.38*** [1.86, 3.04]	3.68*** [2.96, 4.57]	7.05*** [6.03, 8.24]	3.53*** [3.40, 3.67]	1.09 [0.89, 1.33]	1.49*** [1.20, 1.84]	2.92*** [2.36, 3.62]	1.41*** [1.37, 1.45]	1.21 [0.90, 1.62]	1.90*** [1.51, 2.39]	2.32*** [1.94, 2.77]	2.16*** [2.07, 2.24]
40–44	2.02*** [1.56, 2.62]	2.39*** [1.89, 3.02]	3.38*** [2.88, 3.97]	2.12*** [2.04, 2.21]	1.21 [0.98, 1.48]	1.49*** [1.20, 1.85]	2.04*** [1.65, 2.53]	1.27*** [1.24, 1.31]	1.33 [0.98, 1.81]	1.59*** [1.25, 2.02]	2.18*** [1.83, 2.60]	1.81*** [1.74, 1.89]
45–49^a^												
**Years of schooling**												
No education^a^												
< 10 years of schooling	1.01 [0.88, 1.16]	1.28*** [1.12, 1.47]	1.12 [1.01, 1.25]	1.36*** [1.33, 1.40]	0.62*** [0.53, 0.73]	1.13 [0.93, 1.37]	0.77** [0.65, 0.90]	0.82*** [0.80, 0.84]	1.23 [1.01, 1.50]	1.16 [0.97, 1.38]	1.02 [0.88, 1.17]	1.21*** [1.17, 1.24]
≥ 10 years of schooling	1.21 [1.01, 1.45]	1.66*** [1.43, 1.92]	1.24** [1.09, 1.42]	1.40*** [1.36, 1.44]	0.43*** [0.33, 0.55]	0.92 [0.72, 1.17]	0.59*** [0.44, 0.78]	0.55*** [0.54, 0.57]	2.28*** [1.79, 2.90]	1.45*** [1.20, 1.75]	1.41*** [1.16, 1.72]	1.16*** [1.12, 1.19]
**Desire for more children**												
No^a^												
Yes	0.48*** [0.40, 0.56]	0.32*** [0.28, 0.36]	0.48*** [0.43, 0.52]	0.65*** [0.64, 0.67]	0 [0.00, .]	0 [0.00, .]	0 [0.00, .]	0 [0.00, .]	0.76 [0.60, 0.95]	0.35*** [0.30, 0.42]	0.45*** [0.38, 0.54]	0.63*** [0.61, 0.65]
Undecided	0.52*** [0.36, 0.75]	0.34*** [0.29, 0.41]	0.38*** [0.31, 0.48]	0.67*** [0.64, 0.69]	0 [0.00, .]	0 [0.00, .]	0 [0.00, .]	0 [0.00, .]	0.68 [0.41, 1.11]	0.31*** [0.24, 0.40]	0.57** [0.39, 0.84]	0.61*** [0.58, 0.63]
**Gender of living children**												
Only have daughters^a^												
At least have one son	2.06*** [1.78, 2.38]	3.27*** [2.81, 3.80]	2.06*** [1.90, 2.23]	2.09*** [2.05, 2.14]	5.72*** [4.17, 7.85]	6.79*** [3.77, 12.23]	1.89*** [1.52, 2.34]	3.63*** [3.54, 3.72]	1.75*** [1.44, 2.14]	3.66*** [2.95, 4.54]	1.51*** [1.32, 1.74]	1.98*** [1.93, 2.03]
**Mass Media Exposure**												
No^a^												
Yes	1.26** [1.09, 1.47]	1.34*** [1.18, 1.52]	1.16*** [1.06, 1.26]	1.23*** [1.21, 1.26]	1.56*** [1.32, 1.85]	1.41*** [1.16, 1.70]	1.51*** [1.28, 1.78]	1.41*** [1.38, 1.44]	1.56*** [1.22, 1.99]	1.28** [1.08, 1.51]	0.94 [0.83, 1.07]	1.18*** [1.15, 1.21]
**Religion**												
Hindu^a^												
Muslim	0.61*** [0.45, 0.81]		0.62*** [0.55, 0.70]	1.11*** [1.08, 1.14]	0.17*** [0.12, 0.24]		0.58*** [0.47, 0.72]	0.34*** [0.33, 0.35]	0.46*** [0.29, 0.72]		0.62*** [0.52, 0.74]	0.91*** [0.89, 0.94]
Others	1.08 [0.90, 1.30]		1.12 [0.72, 1.76]	0.95*** [0.92, 0.97]	0.59*** [0.45, 0.76]		0.36 [0.11, 1.21]	0.35*** [0.34, 0.36]	0.77 [0.58, 1.01]		1 [0.52, 1.90]	0.87*** [0.84, 0.90]
**Wealth Quintile**												
Poorest^a^												
Poorer	1.18 [1.00, 1.39]	1.51*** [1.27, 1.79]	0.94 [0.84, 1.05]	1.14*** [1.11, 1.17]	2.07*** [1.69, 2.54]	1.34 [1.03, 1.74]	0.84 [0.68, 1.03]	1.29*** [1.26, 1.32]	1.32 [1.03, 1.70]	1.92*** [1.47, 2.51]	1.14 [0.96, 1.36]	1.05** [1.02, 1.08]
Middle	1.13 [0.95, 1.35]	1.89*** [1.57, 2.28]	0.82** [0.73, 0.92]	1.09*** [1.06, 1.12]	2.39*** [1.93, 2.96]	1.61*** [1.21, 2.13]	0.68*** [0.54, 0.86]	1.57*** [1.53, 1.62]	1.16 [0.89, 1.52]	2.94*** [2.23, 3.88]	0.87 [0.72, 1.05]	0.96 [0.93, 0.99]
Richer	1.01 [0.84, 1.21]	1.88*** [1.53, 2.30]	0.84** [0.75, 0.96]	1.17*** [1.13, 1.20]	2.16*** [1.73, 2.70]	1.77*** [1.31, 2.40]	0.65*** [0.51, 0.83]	1.64*** [1.59, 1.69]	1.29 [0.99, 1.69]	3.40*** [2.54, 4.55]	1 [0.82, 1.21]	0.93*** [0.89, 0.96]
Richest	1.18 [0.96, 1.45]	2.16*** [1.73, 2.69]	0.80** [0.69, 0.92]	1.69*** [1.63, 1.75]	2.56*** [1.99, 3.28]	1.78*** [1.28, 2.48]	0.62*** [0.47, 0.81]	1.38*** [1.33, 1.43]	2.21*** [1.67, 2.92]	4.02*** [2.94, 5.49]	0.99 [0.80, 1.23]	1.08*** [1.04, 1.13]
**Place of residence**												
Urban^a^												
Rural	0.92 [0.82, 1.04]	0.93 [0.83, 1.04]	0.72*** [0.66, 0.78]	0.95*** [0.93, 0.97]	0.83** [0.72, 0.95]	1.1 [0.93, 1.31]	0.76*** [0.65, 0.89]	1.17*** [1.15, 1.20]	1.13 [0.96, 1.34]	0.73*** [0.63, 0.84]	0.73*** [0.65, 0.83]	0.93*** [0.90, 0.95]
			Modern Spacing Method AOR (95% CI)			Modern limiting Method AOR (95% CI)			Any traditional Method AOR (95% CI)			
**Region (India)**												
North^a^												
Central			1.42*** [1.37, 1.47]			1.05 [1.01, 1.08]			1.25*** [1.19, 1.31]			
East			0.97 [0.92, 1.02]			0.51*** [0.49, 0.54]			0.69*** [0.64, 0.74]			
Northeast			0.96** [0.93, 0.99]			0.89*** [0.86, 0.91]			1.30*** [1.25, 1.34]			
West			1.10*** [1.07, 1.13]			0.48*** [0.47, 0.50]			1.45*** [1.40, 1.50]			
South			1.04** [1.01, 1.07]			0.67*** [0.65, 0.69]			0.78*** [0.75, 0.81]			
**Region (Nepal)**												
Province 1^a^												
Province 2			0.37*** [0.30, 0.46]			2.46*** [1.94, 3.13]			0.39*** [0.30, 0.52]			
Province 3			1.2 [0.99, 1.47]			1.47** [1.13, 1.91]			0.68** [0.53, 0.89]			
Province 4			0.60*** [0.49, 0.74]			1.04 [0.81, 1.35]			0.59*** [0.46, 0.76]			
Province 5			0.77** [0.64, 0.93]			0.97 [0.76, 1.24]			0.52*** [0.41, 0.66]			
Province 6			0.92 [0.76, 1.13]			1.58*** [1.22, 2.05]			0.50*** [0.38, 0.67]			
Province 7			0.94 [0.77, 1.14]			1.38 [1.08, 1.78]			0.67** [0.52, 0.87]			
**Region (Pakistan)**												
Punjab^a^												
Sindh			0.94 [0.80,1.10]			1.27 [1.03,1.56]			0.63*** [0.51,0.77]			
KPK			1.30*** [1.12,1.52]			0.40*** [0.30,0.52]			0.9 [0.74,1.09]			
Balochistan			0.70*** [0.58,0.86]			0.35*** [0.24,0.49]			0.46*** [0.35,0.61]			
IT			1.16 [0.96,1.41]			0.78 [0.60,1.03]			0.73** [0.57,0.93]			
FATA			1.09 [0.86,1.39]			0.19*** [0.10,0.35]			1.48** [1.12,1.96]			
**Region (Bangladesh)**												
Barisal^a^												
Chittagong			0.66*** [0.57, 0.75]			1.09 [0.79, 1.50]			0.70*** [0.56, 0.86]			
Dhaka			0.95 [0.82, 1.09]			1.79*** [1.31, 2.45]			0.92 [0.74, 1.14]			
Khulna			1 [0.87, 1.15]			1.68** [1.23, 2.30]			1.22 [0.99, 1.50]			
Mymensingh			1.13 [0.98, 1.31]			1.4 [1.00, 1.96]			0.84 [0.66, 1.06]			
Rajshahi			1.1 [0.96, 1.27]			2.09*** [1.54, 2.84]			1.01 [0.81, 1.25]			
Rangpur			1.19 [1.03, 1.37]			2.54*** [1.87, 3.44]			1.26 [1.02, 1.56]			
Sylhet			0.62*** [0.54, 0.72]			1.45 [1.05, 2.01]			0.81 [0.65, 1.02]			

**< 0.01, ***< 0.001

^a^Reference category

Compared to the women living in households comprising husband and/or unmarried child(ren), those from the households comprising husband and other household members excluding MIL had lower odds of using any modern limiting or spacing contraceptive method and any traditional method in all four countries. For example- the women living with a husband and other family members excluding MIL in Bangladesh, had a 30% (OR = 0.70, CI = 0.65–0.76) lesser chance of using any modern spacing method than those staying in households comprising a husband and/or unmarried children. The corresponding figures were 21% (OR = 0.79, CI = 0.70–0.89) for Nepal, 17% for Pakistan (OR = 0.83, CI = 0.75–0.93), and 8% (OR = 0.92, CI = 0.90–0.93) for India.

Similarly, the probability of modern limiting method use was significantly low among women living in households comprising husbands and other family members, excluding the MIL in Bangladesh (OR = 0.81, CI = 0.69–0.94), and India (OR = 0.87, CI = 0.85–0.88). Again, the odds of using any traditional method were significantly low among women living with husband and other family members excluding MIL than those living with husband and/or unmarried child (ren) in Bangladesh (OR = 0.82, CI = 0.73–0.92) and India (OR = 0.92, CI = 0.90–0.94).

Across the countries, the likelihood of using modern spacing and traditional method increased whereas chances of using modern limiting method decreased with increasing years of schooling. The probability of using modern spacing and any traditional contraceptive method was less among those who desire for additional child compared to their counterpart who desire for no more children in all studied countries. Compared to those who had only daughters, those with at least one son had higher likelihood of using any modern as well as traditional contraceptives. The chances to use of any modern as well as traditional contraceptives was higher among women with mass-media exposure than their counterpart with no mass-media exposure. Muslims had less likelihood of using any (except India in modern spacing use) contraceptive method than Hindus in other three countries. In Pakistan, with increasing wealth quintile, the chances of use of all types of contraceptive increases while it decreases in Bangladesh. In Nepal, with increasing wealth quintile, the chances of use of modern limiting method increases in Nepal but in India it leads to use of modern spacing method. Compared to urban women, the chances of contraceptive use among rural women were less except for modern limiting method use in India.

## Discussion

The study found that the living arrangement of women has a significant association with contraceptive use in South Asia. The MIL continues to influence the contraceptive method used by the DIL, albeit country-specific method choice. Co-residence with MIL is associated with higher use of modern spacing method, modern limiting method and traditional method in India, and modern spacing method use in Nepal and Bangladesh. In Pakistan, women living in households without the MIL are less likely to use any contraceptive method.

In Bangladesh, the use of the modern spacing method is more in a household where women live with husbands and other family members, including MIL. There are contradictory findings that have been reported in previous small-scale studies on Bangladesh. A study based on 413 interviews in two areas with different family planning program found contraception use higher among DIL if MIL is supportive [19] while a qualitative explorative study found married adolescents are less likely to use contraception if MIL is against contraception use [28]. Another study based on 4053 currently married women in Matlab Sub district found no influence of the MIL on co-resident DIL’s use of modern contraceptives [29]. In the country, the community health worker network has worked as a backbone to spread the modern spacing method [19, 30] and motivate clients to use contraceptive methods [19, 31]. The results indicate that the community health workers could strengthen the supportive role of the MILs in DIL’s use of the spacing method by gaining trust of MIL through frequent home visits and intensive interactions about the advantages of use of modern spacing methods for birth spacing [19]. We found that any types of contraceptive use is significantly less among women residing in households without MIL, hence the community health worker should identify these households and make extra effort in order to increase the use of contraception among them.

In India, the use of modern limiting methods is more in households with husbands and other family members, including MIL and households with husband and/or unmarried child(ren) and MIL. The results conform to a past study based on data from an earlier round of Indian DHS [32]. Another study reveals that the MILs usually favor the modern limiting method of contraception, influence the timing of use of modern limiting method and oppose the modern spacing method in India [13]. MIL also often force their DIL to prove their fertility in the first year of their marriage, which also restrains the DIL from using any modern spacing method [11, 33]. Studies even indicate that the MIL restricts the mobility of their DIL to disconnect them from the outer world and to maintain their dominance over DIL’s reproductive choice [12, 21]. The use of modern spacing method is high in households with husband and/or unmarried child(ren) and MIL, which may be because the decision to use modern spacing method is majorly taken between husband and wife and thus, though MIL is not in favor of modern spacing method still its use is high. A past small scale qualitative study (Based on 60 MIL, 60 DIL and Sons from same family) conducted in Madhya Pradesh, India conforms this and found that DIL is not comfortable to discuss or disclose about contraceptive use with MIL but they only discuss with husband [13]. Another earlier study also reveal that husband’s decision regarding spacing method use is more important than MIL’s [34]. In the country, the use of any traditional method of contraception is also high among women co-residing with MIL. The result is also in line with a previous study, which reveals that MIL are in favor of traditional methods of contraception for spacing of births [20]. Moreover, the higher use of the traditional method of contraception may be due to less access and knowledge about the modern spacing method among less educated economically poor women or may also be among highly educated wealthy women for the wholesome side effects of the modern contraceptive methods [13, 35].

As women co-residing with MIL are more likely to use modern limiting method and traditional methods, family planning programs may be designed to enhance social networks to increase women’s access and uptake of modern spacing methods. An earlier study also highlights the beneficial role of social networks to increase women’s access and uptake of family planning methods [12]. Again, as MIL often acts as the gatekeeper, future programs may directly target them to inform her about the benefits of family planning [36]. Moreover, schemes like *Saas-Bahu Sammelan* (under Mission Parivar Vikas), a platform for mothers-in-law and their daughters-in-law, which attempts to influence attitudes and ideas about sexual and reproductive health through interactive games and by drawing on personal experiences, needs to be implemented throughout the country. At present this scheme is limited to only 146 high fertility districts (only for rural areas) [37]. A previous study also advocates the beneficial role of this scheme [10].

In Pakistan, contraceptive use is low among women co-residing with other family members without the MIL. Earlier studies from Pakistan also viewed that MIL significantly influences DIL’s contraception use behavior [15, 16]. Evidence further reveals that MIL have greater decision-making authority over young couples’ family planning than either member of the couple itself [38]. Nevertheless, as the overall contraception use is very low in Pakistan compared to other studied South Asian countries, there should be focus on promoting all types of modern contraceptives among women and sensitizing MIL about the benefits of contraceptives by the Lady Health Workers (LHWs) and Family Welfare Assistants (FWAs) placed under existing FP program.

In Nepal, the use of modern spacing is high in a household where women co-reside with MIL. This is in line with previous studies which found that spacing method use can be high through improved and positive spousal communication as well as MIL’s support in delaying childbearing to allow their daughter-in-law to mature, continue her education or earn wages; irrespective of societal pressure upon them [9, 39]. This contradicts to previous literature that reveal MIL as one of the barriers to contraceptive use as she pressurizes DIL to produce a child in the first year itself to prove her fertility, restricts the mobility of DIL, and decides contraceptive use for DIL [17]. Adolescent women are further more vulnerable to the MIL’s decisions on their contraceptive behavior. Nearly one-fifth of the women have unmet need for limiting [40], which has increased in recent times; which suggest the need for expanding the basket of choices in the program to address the need of the women. Additionally, promising interventions such as *Sumadhur* i.e., intervention for triads (wives, husbands, and mothers-in-law) including inequitable gender norms and practices, fertility planning and contraception, and couples and household relationship dynamics, which has been proved beneficial in improving fertility and family planning decision-making norms among newlyweds should be scaled up [41].

Our study found MIL enhances modern spacing method use in Bangladesh, Nepal and India (in set up with only husband and MIL), and influences higher use of modern limiting and traditional methods use in India. Though this study did not analyze whether the method used were informed choices, evidence suggests inadequate informed contraceptive choice in India [42], Bangladesh [43], Nepal [44], and Pakistan [45]. Using contraception without informed choice violates women’s reproductive rights [46]. The inadequately informed choice might lead to higher contraceptive discontinuation. High contraceptive discontinuation rates among women who discontinue use for reasons other than the desire to get pregnant lead to unintended pregnancies [47, 48]. Unintended pregnancies may lead to high maternal morbidity and mortality [49]. Contraceptive discontinuations are further linked with higher unmet needs and induced abortions [50, 51], negatively affecting women’s health.

The study found that irrespective of the studied countries, household living arrangements influence contraceptive method use- contraceptive acceptance is less among women from households with husband and other family members excluding MIL. Co-residence with MIL is associated with higher use of modern spacing method, modern limiting method, and traditional method in India, and modern spacing method use in Nepal and Bangladesh. The results suggest strengthening country-specific family planning programs to ensure informed contraceptive choices in an enabling household environment. Programs proved to improve contraceptive decision making and use among women by addressing concerns of MIL or engaging them in the program may be scaled up to enhance informed contraceptive use.. Irrespective of countries, the use of contraceptives is less among households with husbands and other family members excluding MIL. As an experienced aged female household member, MIL plays a crucial role in DIL contraceptive choice and utilizations, as contraceptives still consider a women’s subject in selected South Asian countries. So, the grassroots-level health workers should identify those households without MIL and give extra attention to them in respect of contraceptive use. This will improve the use of contraceptive use among those households.

The study has some methodological limitations. The study is based on cross-sectional data, thus, limiting any causal association between household living arrangements and contraceptive use. There is a possibility of socio-cultural and contextual factors influencing the contraceptive method use, which this study could not consider due to a lack of data. Despite these limitations, the study’s strengths are that the findings are based on large-scale, nationally representative samples chosen using a robust sampling design. Thus, the results are contemporary and relevant. The results contribute to the existing scanty evidence on the role of MIL in DIL’s contraceptive use in South Asia.

## Conclusion

The study concludes that the living arrangement of the women, especially co-residence with MIL, is associated with the contraceptive method used in South Asia. The MIL continues to influence the contraceptive method used by the DIL, albeit country-specific method choice. The study suggests family planning program to cover MIL for enhancing their understanding on the benefits of contraceptive use and modifying norms around fertility. Strengthening the interaction between the grassroots level health workers and the MIL, enhancing social network of DIL may help informed choice and enhance the use of modern spacing methods. Women’s family planning demands met with modern contraception, and informed contraceptive choices, must also be achieved to reach the 2030 Agenda for Sustainable Development.

## Data Availability

The datasets generated and/or analyzed during the current study are available in the [Demographic and Health Surveys Repository] repository, (https://dhsprogram.com).
